# Diabetic retinopathy and the use of laser photocoagulation: is it cost-effective to treat early?

**DOI:** 10.1136/bmjophth-2016-000021

**Published:** 2017-09-25

**Authors:** Hema Mistry, Peter Auguste, Noemi Lois, Norman Waugh

**Affiliations:** 1 Warwick Medical School, University of Warwick, Coventry, UK; 2 Centre for Experimental Medicine, Queen’s University Belfast, Belfast, UK

**Keywords:** laser photocoagulation, diabetic retinopathy, cost-effectiveness

## Abstract

**Background/aims:**

The aim of the study was to explore whether it would be cost-effective to apply panretinal photocoagulation (PRP) at the severe non-proliferative diabetic retinopathy (NPDR) (early treatment) stage, compared with waiting until high-risk proliferative diabetic retinopathy (HR-PDR) characteristics (deferred treatment) developed.

**Methods:**

A Markov model with a 30-year time horizon was developed, in which patients presenting with moderate NPDR could progress through all stages of DR (severe NPDR>early PDR>HR-PDR>severe PDR) to severe vision loss and blindness (and to death). A National Health Service and personal social services perspective was adopted. Transition probabilities were mainly derived from the Early Treatment Diabetic Retinopathy Study. Health state utilities, costs and complications were based on information from the literature, supplemented by expert opinion. Costs and outcomes were discounted at 3.5%. Both deterministic and probabilistic sensitivity analyses were conducted.

**Results:**

Administering PRP at the severe NPDR stage could be more effective and less costly than waiting until HR-PDR developed. Sensitivity analyses gave similar results, with early treatment continuing to dominate deferred treatment. The probabilistic sensitivity analysis suggests that at willingness-to-pay threshold of £20–£30 000 per quality-adjusted life year, the probability of early treatment being cost-effective is 60%.

**Conclusion:**

PRP administered at the severe NPDR stage is likely to be cost-effective compared with delaying photocoagulation until HR-PDR develops. However, given the limitations of the evidence, these results need to be interpreted with caution. A trial of early versus deferred laser therapy is needed to provide better data based on modern treatments.

**Key messages**

- Panretinal photocoagulation (PRP) is usually administered at the high-risk proliferative diabetic retinopathy (HR-PDR) stage to preserve vision.

- Administering PRP at an earlier stage of retinopathy (severe non-proliferative diabetic retinopathy) could be more cost-effective than delaying PRP until the HR-PDR stage.

- A high-quality research trial of laser treatment at an earlier stage (before HR-PDR develops) in combination with antivascular endothelial growth factor drugs in reducing adverse effects is needed.

## Introduction

Diabetic retinopathy (DR) is a major cause of vision loss in the working age population and people with diabetes are 25 times more likely than the general population to go blind.^1^ Retinopathy can progress through various stages, starting with background (mild) non-proliferative diabetic retinopathy (NPDR), to moderate and severe NPDR, and in some patients to the most serious and sight-threatening form known as proliferative diabetic retinopathy (PDR). If left untreated, this can lead to blindness.

A recent review concluded that rates of progression to PDR have fallen over recent times because of earlier identification and treatment of retinopathy, and improved control of blood glucose and blood pressure.^2^ Nevertheless, DR remains common. A study in Liverpool reported prevalences of any DR and PDR to be 46% and 4% in type 1 diabetes, and 25% and 0.5% in type 2 diabetes, respectively, although the prevalence will vary with mean duration of diabetes, with higher proportions of those with long duration having DR.^3^ However, a more recent study by Yau *et al* found that prevalence for PDR was much higher at 7%.^4^

Scatter or panretinal laser photocoagulation (PRP) is the standard treatment for DR, and in current practice it is administered mostly when DR reaches the high-risk (HR) PDR stage. The aim of PRP treatment is to preserve central vision. However, there are adverse events associated with laser such as visual field defects, retinal fibrosis and epiretinal membrane formation. The ability to drive can also be affected. These harms must be balanced against the benefits of earlier treatment at stages where the risk of blindness is lower. Trials comparing modern lasers with conventional methods have reported little difference in efficacy, but have fewer adverse effects.^5^ In recent years, antivascular endothelial growth factor (anti-VEGF) drugs have become available, and are used for conditions including diabetic macular oedema (DMO). They have to be injected into the eye, but could be used to reduce the risk of DMO associated with PRP.^5^

This paper assesses whether offering PRP treatment to patients with severe NPDR (intervention) could be cost-effective compared with delaying treatment until the HR-PDR stage ensues (usual care).

## Methods

### Base-case analysis

A literature search was undertaken for cost-effectiveness studies of the use of PRP and/or anti-VEGF medication for patients with moderate or severe NPDR. However, we could not identify any appropriate studies.^5^ Therefore, we developed a de novo Markov model in Microsoft Excel to assess the cost-effectiveness of early versus delayed PRP. The different clinical pathways for patients were obtained from information from the Early Treatment Diabetic Retinopathy Study (ETDRS)^6^ and from expert opinion.^5^ The model starts with a hypothetical cohort of 1000 patients with diabetes with a starting age of 50 years presenting with moderate NPDR at an ophthalmology clinic. Fifty years was chosen for the starting age, as this was the mean age of patients with DR.^5^ As DR is a bilateral disease, we have assumed that the model is a two-eye model and that the severity level is the same in each eye. The model assumes that people can progress through all stages of DR, outlined below, but DMO or clinically significant DMO (CSDMO) can occur at any stage:

Moderate NPDR>severe NPDR>early PDR>HR-PDR>severe PDR>severe visual loss/blindness

Online supplementary appendix figures 1–4 show the model structures including the different health states for the intervention and usual care arms. Patients receive treatment and at the end of the cycle they move to the corresponding post-treatment health state. After treatment, patients can progress to a more severe health state, regress to earlier stages, remain as they are or die. The key health states in the model are shown in online supplementary appendix table 1. A 30-year time horizon was adopted with each model cycle length set to 6 months^7^ and transitions between each health state occur at the end of each cycle. A National Health Service (NHS) and personal social services perspective was chosen; a standard annual discount rate of 3.5% was applied to both costs and outcomes. All costs are reported in (£) pounds sterling in 2012/2013 prices. Health outcomes were measured in quality-adjusted life years (QALYs). Results are expressed as incremental cost per QALY gained (incremental cost-effectiveness ratio; ICER).

10.1136/bmjophth-2016-000021.supp1 Supplementary file 1

### Model inputs

Transition probabilities were derived based on information from ETDRS,^6^ as this was the main source of data for the effects of administering PRP at the severe NPDR or early PDR stages rather than waiting until HR-PDR develops (see Royle *et al* for further detailed information on these transition probabilities).^5^ The key transition probabilities are summarised in online supplementary appendix table 2.

Health state utility values were estimated based on a weighted average of two papers (see table 1): Brown *et al* who provided time trade-off values for a range of visual acuities associated with DR^8^ and Fong *et al* who reported the number of people with a range of visual acuity for different stages of DR.^9^ Data from the better seeing eye were used, because quality of life data depends mainly on the better seeing eye. For patients with macular oedema, utility values from a study by Smith *et al* 10 were used. This methodology was similar to a paper by Ting *et al* 11 who developed a Markov model of a novel DR prognostic device for DR progression.^5^

**Table 1:** Health state utility values for the base-case analysis

Health state	Usual care arm	Intervention arm
Moderate NPDR/severe NPDR	0.7915	0.7915
Severe NPDR and CSDMO	0.7365	0.7365
Early PDR/high-risk PDR/severe PDR	0.7047	0.7047
Early PDR and CSDMO/high-risk PDR and CSDMO/severe PDR and CSDMO	0.6930	0.6930
Severe NPDR PT	–	0.7915
Severe NPDR and CSDMO PT	–	0.7365
Early PDR PT	–	0.7047
Early PDR and CSDMO PT	–	0.6930
High-risk PDR PT/severe PDR PT	0.7047	0.7047
High-risk PDR and CSDMO PT/severe PDR and CSDMO PT	0.6930	0.6930
Severe visual loss/blindness	0.6218	0.6218

CSDMO, clinically significant diabetic macular oedema; NPDR, non-proliferative diabetic retinopathy; PDR, proliferative diabetic retinopathy; PT, post-treatment.

Resource use information was based on information from the Royal College of Ophthalmologists guidelines^7^ and from our own experience (NL). During each 6-month cycle, the number of ophthalmology and monitoring visits was conservatively estimated as follows: for patients with moderate or severe NPDR they would have one visit, for patients with early PDR they would average 1.5 visits, for patients with HR-PDR or severe PDR they would have two visits, and for patients with severe vision loss/blindness they would have half a visit (ie, one visit per year). For patients receiving PRP treatment, we have assumed that both eyes will be treated at the same time and PRP treatment will be given over two sessions to reduce the risk of DMO. Patients who also have DMO will receive focal laser first for both eyes, and an optical coherence tomography test would also be undertaken. The majority of unit costs were obtained from the NHS reference costs database^12^ (see table 2).

**Table 2:** Unit costs for the base-case analysis

Resource use (HRG code)	National average unit cost	Source
Ophthalmology clinic visit (WF01B)	£106	12
Monitoring clinic visit (WF01A)	£80	12
PRP laser (OP BZ22B)	£131	12
Focal laser (OP BZ22B)	£131	12
Optical coherence tomography (OP BZ23Z)	£117	12
Vitrectomy surgery (DC BZ22B)	£989	12
Annual cost of blindness*	£1483	26

*Excludes residential (home) care.

HRF, Healthcare Resource Group; PRP, panretinal photocoagulation.

Some patients who receive PRP may also develop complications. We have assumed for one cycle only that a proportion of people who receive PRP will develop macular oedema^6^ or less often, vitreous haemorrhage (in patients who have severe PDR/severe PDR and CSDMO).^13^ For this cycle, we have included the appropriate treatment cost and a disutility value of −0.03.^14^

Age-specific mortality rates used in the model were derived from the UK general population lifetime tables from the Office of National Statistics.^15^ People with diabetes have about twofold higher mortality than people without diabetes^16 17^—mortality multiplier of 2.194 was applied in the model; and people with diabetes and advanced retinopathy have higher mortality than people with diabetes but no advanced retinopathy.^18 19^ We therefore applied four mortality multipliers depending on severity: moderate NPDR=1.118, severe NPDR=1.422, mild PDR=0.992 and moderate/high PDR=1.705.

### Sensitivity analysis

To take account of uncertainties in the parameters used in the model and to illustrate sampling uncertainty, we ran the model deterministically and probabilistically with 1000 iterations. These bootstrapped iterations were plotted onto cost-effectiveness planes and they were also used to calculate the cost-effectiveness acceptability curves (CEACs). CEACs were presented using a willingness-to-pay (WTP) threshold from £0 to £50 000. For the probabilistic analysis, the gamma distribution was used for costs and the beta distribution was used for utility values. Various sensitivity analyses were conducted by altering base-case inputs to the model; including a one-way sensitivity analysis (tornado diagram) where a number of key variables were varied holding others constant to understand what was influencing the net monetary benefit of PRP treatment.

## Results

**Figure 1:**
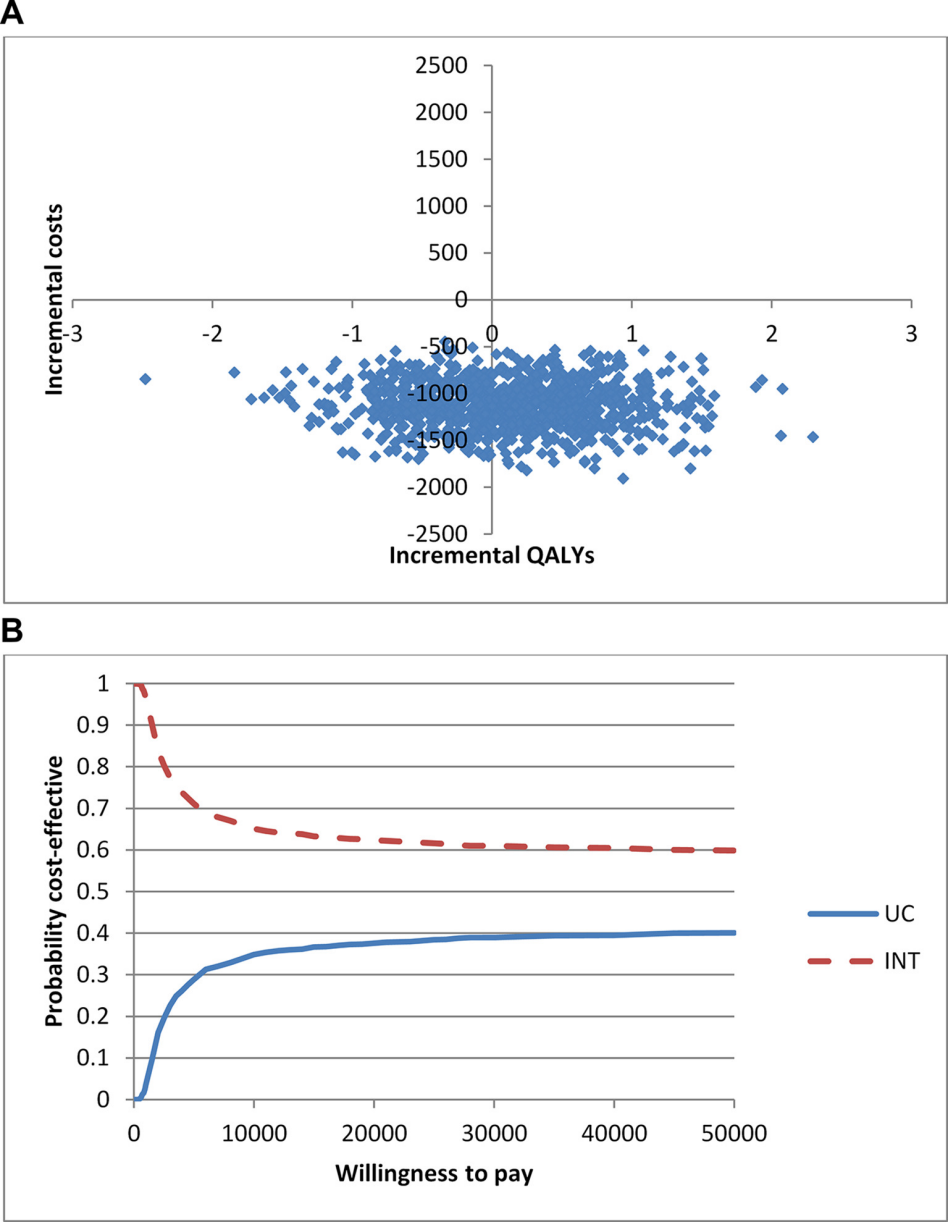
(A) Cost-effectiveness plane—usual care (usual care) versus intervention (early PRP). (B) Cost-effectiveness acceptability curve—usual care (usual care) versus intervention (early PRP). INT, intervention; PRP, panretinal photocoagulation; QALYs, quality-adjusted life years; UC, usual care.

In the base-case analysis, if PRP treatment were delayed until HR-PDR developed, this was more costly and less effective than if PRP treatment were administered to patients with severe NPDR (see table 3). Treating earlier with PRP laser meant that fewer people in the intervention arm compared with the usual care arm progressed to more advanced stages of DR. The uncertainty in the results is shown in the cost-effectiveness plane (figure 1A). If a decision maker is willing to pay between £20 000 and £30 000 per QALY, there is a 60% probability that early PRP is more cost-effective than usual care (figure 1B).

**Table 3:** Base-case cost-effectiveness results (discounted)

	Usual care (PRP at HR-PDR)	Intervention (early PRP at severe NPDR)
*Deterministic*		
Total mean costs (£)	£3853	£2753
Total mean QALYs	7.8236	7.9572
Incremental costs (£)/QALYs	−£1101/0.1337	
ICER (cost per QALY gained)	Dominated	
*Probabilistic*		
Total mean costs (£)	£3858	£2746
Total mean QALYs	7.8332	7.9624
Incremental costs (£)/QALYs	−£1112/0.1292	
ICER (cost per QALY gained)	Dominated	

table 4.

HR-PDR, high-risk proliferative diabetic retinopathy; ICER, incremental cost-effectiveness ratio; NPDR, non-proliferative diabetic retinopathy; PRP, panretinal photocoagulation; QALYs, quality-adjusted life years.

Overall, the results from the deterministic sensitivity analyses (Table 4) are in line with the base-case analysis where the intervention dominates usual care. That is, early PRP is still cheaper and more effective than delayed PRP. When we assumed that patients would receive PRP treatment in the intervention arm in the early PDR stages as opposed to the severe NPDR stages, the cost and QALY differences between the two arms were negligible. This may be due to the source of progression data used in the model, as the ETDRS did not report results separately for patients with severe NPDR and early PDR.

**Table 4:** Deterministic sensitivity analysis cost-effectiveness results

	Usual care (usual care)	Intervention (early PRP)
*Patients in the intervention arm receive PRP treatment at the **early** PDR or early PDR and CSDMO instead of the severe NPDR or severe NPDR or CSDMO stage.*		
Total mean costs (£)	£3853	£3725
Total mean QALYs	7.8236	7.8645
ICER (cost per QALY gained)	Dominated	
*PRP treatment is administered in one sitting (two laser treatments for two eyes) or in four sittings (eight laser treatments for two eyes) instead of two sittings (four laser treatments for two eyes). We have assumed that the risk of DMO remains the same.*		
*One sitting*		
Total mean costs (£)	£3762	£2452
Total mean QALYs	7.8236	7.9572
ICER (cost per QALY gained)	Two sittings are dominated by one sitting	
*Four sittings*		
Total mean costs (£)	£4035	£3353
Total mean QALYs	7.8236	7.9572
ICER (cost per QALY gained)	Two sittings are dominated by four sittings.	

CSDMO, clinically significant diabetic macular oedema; DMO, diabetic macular oedema; ICER, incremental cost-effectiveness ratio; NPDR, non-proliferative diabetic retinopathy; PDR, proliferative diabetic retinopathy; PRP, panretinal photocoagulation; QALYs, quality-adjusted life years.

**Figure 2:**
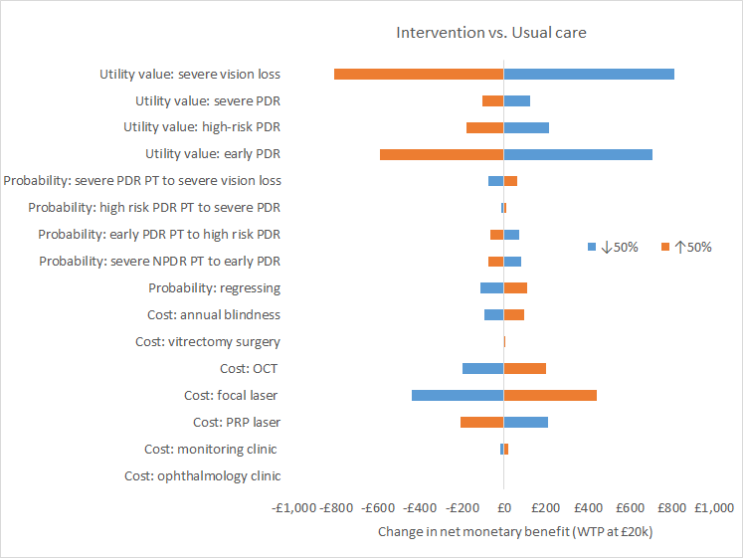
Tornado diagram for net monetary benefit usual care (usual care) versus intervention (early PRP). NPDR, non-proliferative diabetic retinopathy; OCT, optical coherence tomography; PDR, proliferative diabetic retinopathy; PRP, panretinal photocoagulation; PT, post-treatment; WTP, willingness to pay.

Figure 2 is centred around the net monetary benefit ratio of £3774 for intervention when compared with usual care with a WTP threshold set at £20 000. The most important variables influencing the net monetary benefit are the utility values for severe vision loss and early PDR. For the intervention arm, increasing the utility value for severe vision loss by 50% (utility value is capped at 1.00), the net monetary benefit ratio falls to £2964, whereas reducing the utility value for severe vision loss by 50% the net monetary benefit ratio increases to £4584. Other influences include the costs of the focal and PRP lasers.

## Discussion

A thorough search for cost-effectiveness studies of the use of PRP and/or anti-VEGF medication for patients with severe NPDR or early PDR found no economic evaluations or modelling-based studies with which to compare our results.

In the present study, we built a Markov model to assess whether offering PRP treatment to patients with severe NPDR (intervention) is cost-effective compared with delaying treatment until the HR-PDR stages (usual care). We found that treating earlier at the severe NPDR stage was more cost-effective than delaying treatment until HR-PDR stages. To check the inherent uncertainty in the base-case results, various sensitivity analyses were undertaken and the majority of results were in line with the base-case analyses.

The strengths of the study are: (1) to our knowledge, this is the first model that compares the cost-effectiveness of using PRP treatment at an earlier stage of DR (severe NPDR) as opposed to current practice (HR-PDR); and (2) it contains more detailed health states differentiated by the different severity levels for DR.

However, the evaluation has various shortcomings. First and most notably is the use of progression data mainly from the ETDRS trial.^6^ More recent studies have been conducted but they have not addressed the issue of early versus delayed PRP (timing). Due to improved treatments, better blood glucose control and population screening, monitoring and intervention, there has been a reduction in the incidence of severe vision loss/blindness from DR.^2 4^ Second, even though we conducted a thorough search of the literature, we did not identify any studies with health state utility values by the detailed severity levels that we have in our model or data on disutilities associated with progressing through all the different stages of DR, or data on the disutilities after PRP, especially following the use of modern laser delivery devices. We had to rely heavily on two papers^9 10^ to characterise the different visual acuity levels into health states and link them to the utility values for patients with DR as reported in Brown *et al*.^8^ The key limitation from this methodology was the amalgamation of DR stages, that is, we have the same utility value for a patient with early PDR as someone who has severe PDR,^9^ and we did not know whether macular oedema was clinically significant or not, and whether there was any visual impairment.^10^

Third, we have assumed that the cost of PRP is for a generic PRP laser machine as we did not have costs for PRP with all types of laser machines. Also, we have assumed that the unit costs of PRP and focal laser are the same, even though PRP takes much longer and requires more sessions than focal laser. Even if we had more accurate costs by carrying out ‘bottom-up costing’, we do not believe that this would have made a difference to the ICER.

Fourth, PRP destroys retinal tissue, and this may lead to symptoms due to the loss of function of the burned areas, including peripheral visual field defects, reduced night vision and decreased contrast sensitivity. A systematic review by Fong and colleagues reported that visual field defects could occur in up to 50% of treated patients, depending on the intensity of PRP and the level of testing using different isopters.^20^ They also noted that after PRP, 38% of people reported worsened night driving and 60% worsened dark adaptation. Preti and colleagues reported deterioration in contrast sensitivity after PRP, but giving intravitreal bevacizumab prevented this deterioration.^21^ The model will not have explicitly captured the costs and effects of any of these adverse visual field effects.

One problem with studies of adverse effects of PRP is that the data come from studies in patients with HR-PDR. If PRP was given at the severe NPDR stage, it is likely possible to reduce the amount of treatment given (compared with what is required to treat HR-PDR) with an expected reduction in adverse effects.

In the model, we have assumed that a proportion of people develop adverse events after laser treatment such as DMO and vitreous haemorrhage. However, these complication rates were based on the ETDRS studies^6^ which may no longer be applicable given that the development of CSDMO following PRP when performed with current protocols and with modern laser technologies appears to be less frequent.^5^ In the economic model, we have not included a cost or disutility for pain after PRP (although costs for a simple analgesic such as paracetamol are negligible), nor for any adverse events (such as scotomas) due to focal laser.^22^ However, we believe that this would not alter the magnitude and direction of the ICER because adverse effects would occur whether PRP was early or deferred. Another limitation is that PRP might be administered to some people with NPDR who would not have progressed, so we might treat more people not all of whom would benefit.

Trials have been done or are being undertaken comparing anti-VEGF treatment such as ranibizumab, compared with PRP for the treatment of PDR. At 1 year, ranibizumab treatment was non-inferior to PRP, but presumably at a much higher cost because of the number of injections required, making the cost-effectiveness doubtful.^23^ We are not aware of any trials of anti-VEGF therapy versus PRP in severe NPDR.

Finally, we have not differentiated whether the patient had type 1 or type 2 diabetes. The ETDRS reported better results with early intervention (PRP) in type 2 diabetes. So, the key uncertainties are:

- differences in effectiveness and adverse events between the argon laser-based PRP used in ETDRS and the new laser technologies used today;
- the quality of life effects using a finer gradation of DR severity;
- the disutilities of PRP;
- whether progression is now reduced or slower than was seen in ETDRS;
- differences between type 1 and type 2 diabetes;
- whether earlier PRP would be given at severe NPDR or early PDR stage.

Our analysis suggests that earlier PRP could be cost-effective, but there are too many uncertainties to make a firm recommendation. Even though PRP might be cost-effective for the overall population for whom the treatment is intended, there may be occasional individuals for whom the treatment would be harmful in the short term. For example, a patient with good visual acuity but severe NPDR might lose the capability to work or drive due to peripheral visual loss caused by PRP.

We suggest that a trial is needed that compares early versus deferred PRP with modern laser methods and devices, and also assesses the role of anti-VEGF or steroid drugs in combination with PRP in reducing new DMO or as adjuvant in those with pre-existing DMO. An economic evaluation alongside the trial would collect accurate cost estimates for the different regimens and also collect detailed health-related quality of life measures to enable calculation of QALYs. As generic-based measures such as EQ-5D are said to be insensitive to the changes in visual impairment due to DR progression,^24^ the trial should also include a disease-specific measure such as NEI-VFQ-25 questionnaire.^25^

## Conclusions

Our economic model suggested that PRP administered at the severe NPDR stage might be cost-effective. However, given the limitations of the evidence on current treatments, these results should be interpreted with caution. A trial of early versus deferred laser therapy (with or without anti-VEGF medication) is needed to fully answer the question.
