# Supplementary material for: Diabetic retinopathy and the use of laser photocoagulation: is it cost-effective to treat early?
Source: BMJ Open Ophthalmol. 2017 Sep 25;2(1):e000021. doi: 10.1136/bmjophth-2016-000021 (PMC5721644; doi:10.1136/bmjophth-2016-000021)
Supplement: Supplementary file 1 [file bmjophth-2016-000021supp001.docx]

Appendix: Figure 1 - Current practice (usual care) progression

Appendix: Figure 2 - Current practice (usual care) regression

Appendix: Figure 3 - Early PRP (intervention) progression

Appendix: Figure 4 - Early PRP (intervention) regression

Key: CSDMO, clinically significant diabetic macular oedema; NPDR, non-proliferative diabetic retinopathy; PDR, proliferative diabetic retinopathy; PT, post-treatment; VI, visual impairment

**Appendix Table 1: Health states in the model**

| ***Health state*** | ***Post-treatment health state*** |
| --- | --- |
| Moderate NPDR |  |
| Severe NPDR | Severe NPDR PT |
| Severe NPDR and CSDMO with/without VI | Severe NPDR and CSDMO with/without VI PT |
| Early PDR | Early PDR PT |
| Early PDR and CSDMO with/without VI | Early PDR and CSDMO with/without VI PT |
| High-risk PDR | High-risk PDR PT |
| High-risk PDR and CSDMO with/without VI | High-risk PDR and CSDMO with/without VI PT |
| Severe PDR | Severe PDR PT |
| Severe PDR and CSDMO with/without VI | Severe PDR and CSDMO with/without VI PT |
| Severe visual loss/blindness |  |
| Death |  |

A Markov (state-transition) model was developed using Microsoft Excel. Model structure for people receiving current practice (usual care) is shown in Figures 1 and 2 and the model structure for people receiving early PRP (intervention arm) is shown in figures 3 and 4. Health states (HS) in the model structure are shown in the ovals, the arrows represent the transitions that patients can make in the model (patients can either progress or regress), the recurring arrows show that patients can stay in that same health state for more than one cycle, and death is an absorbing health state. Patients can die from diabetes related causes or die from other causes.

The model starts with patients presenting with moderate NPDR and assumes that people progress through all stages of diabetic retinopathy:

Moderate NPDR ⇨ severe NPDR ⇨ early PDR ⇨ high-risk PDR ⇨ severe PDR

In the first cycle, patients stay in the moderate NPDR HS or they progress to either severe NPDR HS or to severe NPDR and CSDMO with/without VI HS or die. In the intervention arm, those patients in the severe NPDR HS or the severe NPDR and CSDMO with/without VI HS, receive treatment and at the end the cycle they move to the corresponding post-treatment HS.

Usual care arm: In the second cycle, patients can stay in either the moderate or severe NPDR HS or progress to early PDR or early PDR and CSDMO with/without VI or die. In the next cycle, the patients can stay in the moderate NPDR, severe NPDR or early PDR HS or progress to high-risk PDR or high risk PDR and CSDMO with/without VI HS or die (patients in the early PDR and CSDMO with/without VI can also progress to the severe visual loss/blindness HS because of DMO). Once the patient moves to the high-risk PDR or high risk PDR and CSDMO with/without VI HS, they receive treatment and at the end the cycle they move to the corresponding post-treatment HS. Once the patients enter the post-treatment HS, they can either stay in this HS or progress to one of the more severe HS, regress back to earlier stages of the disease, or die.

Intervention arm: In the second cycle and onwards, patients can stay in the moderate NPDR HS or progress to either severe NPDR or severe NPDR and CSDMO with/without VI or die. For those patients who were in the severe NPDR HS in the second cycle, in the third cycle they can progress to early PDR or early PDR and CSDMO with/without VI and so forth. When the patients progress to one of these HS (i.e. severe NPDR or early PDR) they will receive treatment and at the end the cycle they move to the corresponding post-treatment HS. Patients who received treatment in the previous cycle, they start in the post-treatment HS and they can either stay in this HS or progress to one of the more severe HS, regress back to earlier stages of the disease, or die.

Both treatment arms: Patients can stay in the post-treatment health state for more than one cycle.

Appendix Table 2: Key transition probabilities

|  | **Moderate NPDR** | **Severe NPDR** | **Early PDR** | **High risk PDR** | **Severe PDR** | **Severe visual loss** |
| --- | --- | --- | --- | --- | --- | --- |
| **For the progression of diabetic retinopathy (intervention and usual care)** | | | | | | |
| **Moderate NPDR** | 0.94981 | 0.0221 | 0 | 0 | 0 | 0 |
| **Severe NPDR** | 0 | 0 | 0.0214 | 0 | 0 | 0 |
| **Early PDR** | 0 | 0 | 0 | 0.0717 | 0 | 0 |
| **High risk PDR** | 0 | 0 | 0 | 0 | 0.0459 | 0 |
| **Severe PDR** | 0 | 0 | 0 | 0 | 0 | 0.0535 |
| **Severe visual loss** | 0 | 0 | 0 | 0 | 0 | 1.0000 |
| **For the progression of diabetic retinopathy (usual care) - post treatment** | | | | | | |
| **High risk PDR PT** | 0.0036 | 0.0036 | 0.0036 | 0 | 0.0459 | 0 |
| **Severe PDR PT** | 0.0036 | 0.0036 | 0.0036 | 0.0036 | 0 | 0.0252 |
| **For the progression of diabetic retinopathy (intervention) - post treatment** | | | | | | |
| **Severe NPDR PT** | 0.0036 | 0 | 0.0171 | 0 | 0 | 0 |
| **Early PDR PT** | 0.0036 | 0.0036 | 0 | 0.0186 | 0 | 0 |
| **High risk PDR PT** | 0.0036 | 0.0036 | 0.0036 | 0 | 0.0459 | 0 |
| **Severe PDR PT** | 0.0036 | 0.0036 | 0.0036 | 0.0036 | 0 | 0.0252 |

Please note each row may not add up to 1 as not all health states have been included in the table
